# Mesoporous biophotonic carbon spheres with tunable curvature for intelligent drug delivery

**DOI:** 10.1515/nanoph-2022-0523

**Published:** 2022-10-04

**Authors:** Jianye Fu, Tiankun Hui, Dong An, Wei Shan, Guobo Chen, Swelm Wageh, Omar A. Al-Hartomy, Bin Zhang, Ni Xie, Guohui Nie, Jinqing Jiao, Meng Qiu, Han Zhang

**Affiliations:** Key Laboratory of Marine Chemistry Theory and Technology (Ocean University of China), Ministry of Education, Qingdao, 266100, China; Collaborative Innovation Center for Optoelectronic Science & Technology, International Collaborative Laboratory of 2D Materials for Optoelectronics Science and Technology of Ministry of Education, College of Physics and Optoelectronic Engineering, Institute of Microscale Optoelectronics, Shenzhen University, Shenzhen 518060, P. R. China; College of Chemistry and Chemical Engineering, China University of Petroleum, Qingdao 266555, China; Department of Physics, Faculty of Science, King Abdulaziz University, Jeddah 21589, Saudi Arabia; State Key Laboratory of Safety and Control for Chemicals, SINOPEC Research Institute of Safety Engineering, Qingdao, 266071, China

**Keywords:** biophotonic carbon spheres, controlled release, drug delivery, NIR photoresponse, pore structure modulation

## Abstract

Mesoporous carbon spheres (MCSs) are widely used in the field of pollutants adsorption, energy storage and various biomedicine applications such as targeted delivery vector, phototherapy sensitizers, bioimaging contrast agents, etc. Current synthetic strategies including soft templating and hard templating methods generally have the limits of using expensive surfactants or lack of control over the pore structures. Therefore, the complex and uncontrollable pore structures limit its further clinical application. Herein, we proposed a new synthetic strategy to control the uniformity of pore channel arrangement in MCSs which can modulate the photonic property and the corresponding light controlled drug release property in intelligent drug delivery. The as obtained MCSs with relative uniform pore channel arrangement and long pore channels are demonstrated to have the best NIR light-induced drug release performance. This work provides not only new synthetic method to modulate pore structure characteristics and biophotonic property of MCSs, but also uniform MCSs as novel delivery platforms with advanced controlled release performance.

## 1 Introduction

Recently, research on the fabrication of mesoporous carbon spheres (MCSs) has attracted increasing attention [1–7]. The outstanding properties of MCSs such as controlled structures, adjustable pore size, large surface area and good adsorption capability has made them ideal candidates for various applications [8–12]. For example, when MCSs are used as drug delivery vectors, they can avoid the inherent drawbacks brought by the widely used mesoporous silica materials, such as weaken the immune system, granulation in the pulmonary and tracheobronchial lymph nodes [13, 14]. However, the complex and uncontrollable pore structures limit its further clinical intelligent drug delivery applications. It is believed that the uniformity of pore channel arrangement and the length of pore channels in MCSs can significantly influence its photonic properties as well as their potential applications. Moreover, when materials with uniform arranged pore channels are applied as carriers for controlled drug delivery, it is widely believed that the uniformity of pore channels are strongly related with the matrix and drug interactions that determine the adsorption, mass transport and release behaviors of loaded drugs. Therefore, mesoporous materials with uniform arranged pore structures are highly demanded [15–19].

A variety of fabrication methods have been reported to synthesize MCSs with relative uniform arranged pore structures, such as soft templating method, hard templating method, etc. [8, 20] The soft templating fabrication method usually involve the use of surfactants such as Pluronic P123, F127, CTAB and SDS [21–23]. However, the use of surfactants in soft templating fabrication method has its own drawbacks; for example, these toxic and expensive surfactants significantly restricted their large-scale manufacturing [24]. Moreover, the surfactant residue may cause irreversible adsorption on the surface of nanoparticles, which lead to the reduced amount of accessible adsorption sites and other undesired properties [25]. In addition to the soft templating method, the hard templating method as a surfactant-free strategy to prepare MCSs can avoid the drawbacks brought by soft templating method [8, 26, 27]. While the structure of the as obtained MCSs rely closely on the structure of the hard templates, which indicate the corresponding pore structure characteristic are difficult to control and lack of variety, especially the uniformity of pore structures. Though the hard templating method does not need the presence of surfactants, it requires pre-synthesized templates and multiple steps, which increase the synthesis difficulty [28]. Therefore, it is still a great challenge to easily control the diversity of the hard templating method and fabricate MCSs with tunable pore structures, as well as the surfactant-free fabrication of MCSs with relative uniform arranged mesoporous structures.

**Scheme 1:**
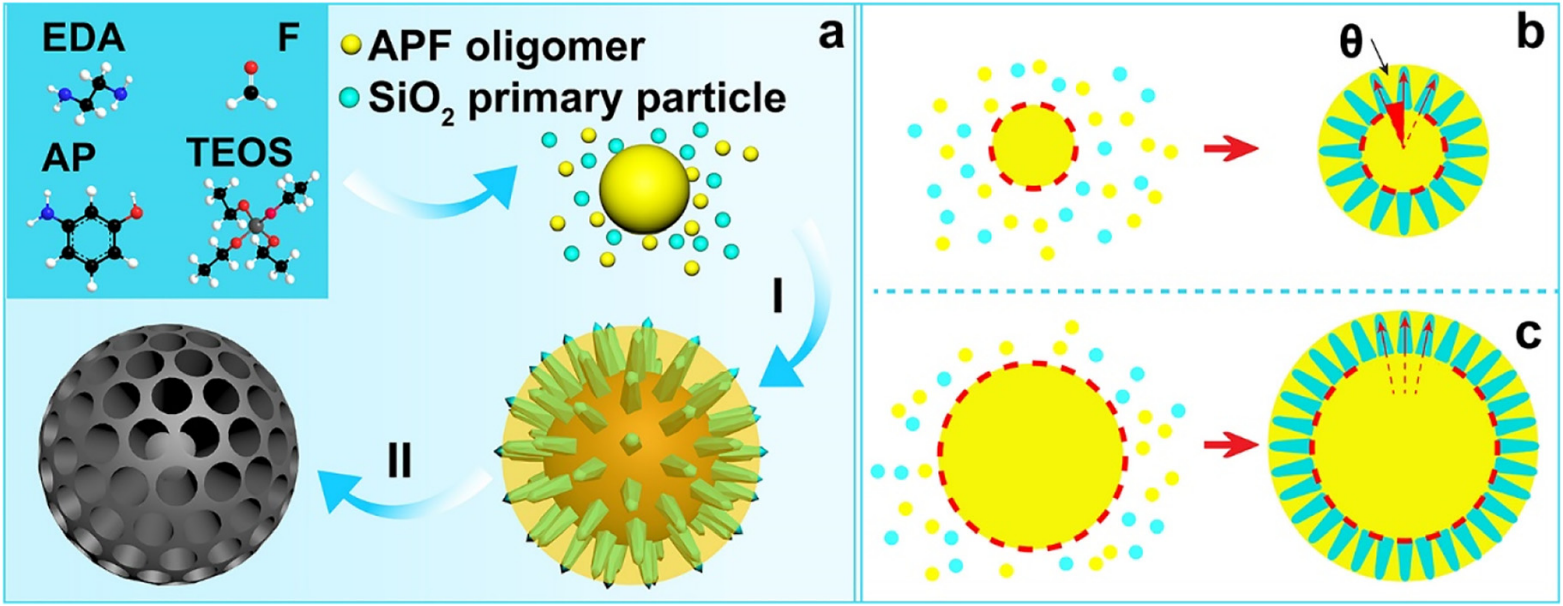
The reaction kinetic controlled surfactant-free synthesis approach of MCSs (a), co-condensation process (I), carbonization in N_2_ atmosphere and etching of silica template (II); growth behavior of the silica spikes on APF cores, central radial behavior (b) and para-parallel behavior (c).

Herein, we develop a curvature assisted strategy to adjust the pore channel arrangement of MCSs for the first time. The new strategy involves a reaction kinetic controlled surfactant-free synthesis approach by using 3-aminophenol and formaldehyde as the carbon source, *in situ* generated silica primary particles as the hard templates, ethanol and water as a mixed solvent and ammonia as the reaction catalyst (Scheme 1). Since the structure of silica template and the pore channels of MCSs are counterpart to each other, the porous nature of MCSs is determined by the present of the *in situ* generated silica hard templates. Through simply adjusting the reaction kinetic of the two polymerization species, curvature of the core particles can be easily controlled, which can be used to assist the control of growth behavior of silica spike structure and the corresponding uniformity of pore channels (Scheme 1b and c). At last, we demonstrate the potential application of using MCSs as an outstanding drug delivery system with NIR light response to control the release of loaded cargo molecules, and the MCSs based drug delivery platform exhibit advanced cancer cell killing efficacy. The uniform arranged mesoporous structure has been shown related with the photothermal capability and the release performance of the MCSs. We believe the new developed synthetic strategy has paved the way to control the uniformity of pore channel characteristics through surfactant-free approaches. Moreover, the as prepared MCSs can be used as versatile platforms for various applications.

## 2 Results and discussion

**Figure 1:**
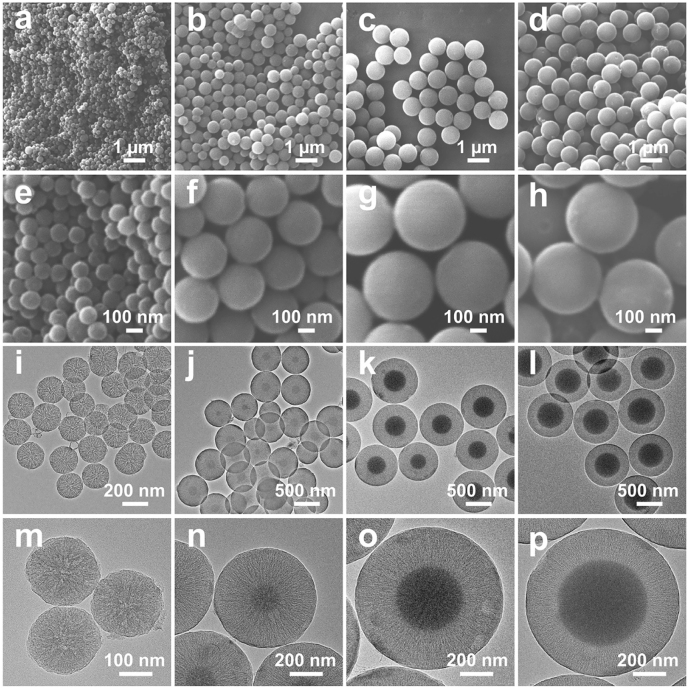
SEM images (a–h) and TEM images (i–p) of MCS-1 (a, e, i, m), MCS-2 (b, f, j, n), MCS-3 (c, g, k, o), and MCS-4 (d, h, l, p).

Uniform MCSs with various pore structure characteristics are obtained through a water-ethanol solution with 3-aminophenol and formaldehyde as precursors and silica as *in situ* generated hard template. The pore structure characteristics of MCSs are strongly related with the configuration of the *in situ* generated silica hard template, which can be modulated by the delay addition of silica precursor (TEOS). With the increase of delay addition time interval, APF polymer has prolonged polymerization time to generate larger core size particles with small curvature for the following deposition of silica primary particles. The MCSs are named as MCS-1, MCS-2, MCS-3, and MCS-4 for time intervals of 1 min, 2 min, 3 min, and 10 min, respectively (see experimental section for more details). During the synthesis of MCSs, APF/silica composite spheres are firstly obtained as intermediate products. As shown in Figure S1, APF/silica composite spheres exhibited uniform nanoparticle size and well dispersed property, the size of which increased with the time interval of delay addition. After carbonization and silica etching of the APF/silica composite spheres, MCSs are obtained accordingly. Scanning electron microscopy (SEM) characterizations were used to evaluate the structural properties of MCSs. As shown in Figure 1, MCS-1 (Figure 1a and e) have uniform particles size of ∼200 nm, with the increase of the time interval, the particles size of MCSs increases to ∼500 nm for MCS-2 (Figure 1b and f), ∼800 nm for MCS-3 (Figure 1c and g) and MCS-4 (Figure 1d and h).

Moreover, transmission electron microscope (TEM) images confirm that MCSs have uniform particles sizes, consistent with the observation from SEM images. The porous characteristic of MCSs can be clear seen from the TEM images, for example, MCS-1 exhibit a central radial pore channels where the pore channels interconnect with each other and extend from the inner center of the nanoparticle to the outer surface (Figure 1i and m). With further increase the delay addition time interval to 2 min (MCS-2, Figure 1j and n), it is interesting to notice that a carbon core with size of ∼100 nm emerges in the nanoparticles. Moreover, the core size increases with the delay addition time interval and reach to ∼500 nm for MCS-4 (delay addition time interval of 10 min). Furthermore, as shown in the high resolution TEM images (Figure 1m–p), the pore channels change from irregular in MCS-1 (Figure 1m) to gradually uniform arranged mesoporous structures in MCS-3 (Figure 1o) and MCS-4 (Figure 1p), indicating the pore channel growth behavior changed from the central radial behavior to a para-parallel behavior. The change of pore channel growth behavior is related with the change of the curvature of core particles, i.e., MCS-1 with the smallest core size (negligible to observe, Figure 1m) have the largest core curvature, which result in the central radial growth of silica spike as templates and the corresponding central radial pore channels of MCS-1. Moreover, MCS-3 and MCS-4 with the largest core sizes have the smallest core curvature that result in the para-parallel alignment of silica spikes and corresponding para-parallel pore channels of MCS-3 and MCS-4. The above observation confirms that the porous characteristics of MCSs are successfully controlled by the curvature of core particles, and with the decrease of core curvature, more uniform pore channels can be prepared.

**Figure 2:**
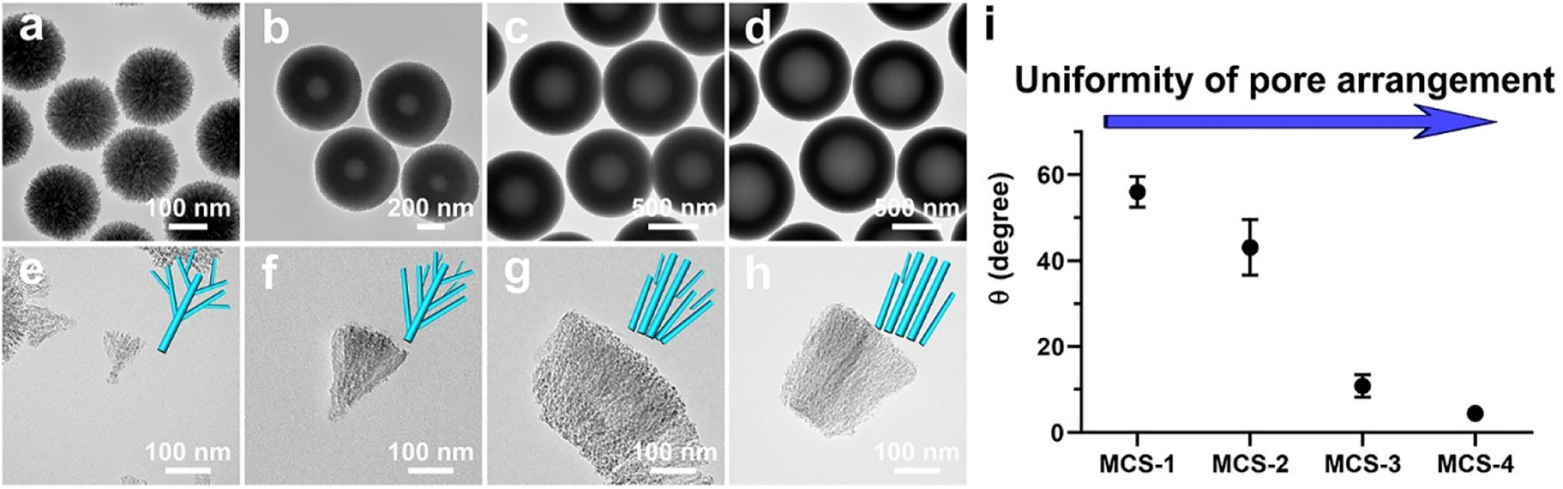
TEM images of the silica templates (a, b, c, d) and their corresponding deprived fragments (e, f, g, h) of MCS-1 (a, e), MCS-2 (b, f), MCS-3 (c, g) and MCS-4 (d, h); measured angles between adjacent spikes in silica templates from corresponding MCSs (i).

The structures of the *in situ* generated silica templates were also prepared and characterized to demonstrate the above analysis of pore structure modulation strategy. The as-synthesized silica/APF polymer composites of each MCSs were calcined to remove APF polymer and the silica templates were obtained accordingly. The silica templates and their related fragments were examined by TEM. As shown in Figure 2a–d, it can be seen that the silica templates have similar sizes compared with that of MCSs, respectively. Moreover, a hollow cavity can be clearly observed in the template of MCS-2 and the hollow cavity grows larger along with the increase of the delay addition time interval (MCS-3 and MCS-4). Furthermore, it is seen that all the silica templates are consisted by spike-like structures. Curvature of the cores in silica templates decreased from MCS-1 to MCS-4, and the curvature can influence the growth behavior of the silica spikes accordingly. For example, for cores with high curvature (MCS-1), the growth of silica spikes follows a central radial growth pattern, and the spikes generally interact with the adjacent ones. While for cores with low curvature, the perpendicular growth of the silica spikes follows a para-parallel pattern and result in relative uniform arranged pore structures, where the silica spikes have no interaction with the adjacent ones. As shown in Figure 2, the central radial growth behavior can be clearly observed for the silica spike templates of MCS-1 (Figure 2e) and MCS-2 (Figure 2f). The alignment of silica spikes become relative uniform and the growth behavior changed to para-parallel behavior for MCS-3 (Figure 2g) and MCS-4 (Figure 2h). Since the silica templates are counterparts of the MCSs, the hollow cavity of the silica templates indicates the presence of solid cores in MCSs and the spike-like structure suggest the characteristic of the pore channels, which is consistent with the previous characterization of MCSs. Furthermore, uniformity of arrangement of the mesopore channels is characterized and the angle is measured between adjacent silica spikes in silica templates, which represent the structure of pore channels in MCSs. As shown in Figure 2i, results show that the angle decreased from MCS-1 to MCS-4, which further indicate that the pore channels are relative uniform arranged and become para-parallel in MCS-4. Therefore, the delay addition strategy can successfully modulate the uniformity of pore channels in MCSs through the curvature assisted surfactant-free synthesis mechanism.

Based on the above observations and discussions, we propose a curvature assisted surfactant-free synthesis mechanism to explain the control strategy over the uniformity of pore channels. During the synthetic process, 3-aminophenol (AP) and formaldehyde (F) first polymerize to generate APF oligomers, which condense into APF polymer core particles. Afterwards, TEOS starts to hydrolyze and generate silica species/silica primary particles, which co-condense with APF oligomers. The generated silica species/silica primary particles functions as *in situ* hard templates (Scheme 1). The heterogeneous nucleation of silica primary particles on APF core particles form isolated islands which limit the spherical homogeneous condensation of APF oligomers. Then the following condensation of silica primary particles and APF oligomers preferentially occur on the previous generated silica islands and APF matrix, respectively (Scheme 1I). As a result, the growth of silica template follows by a central radial behavior and form silica spike structures perpendicularly on the APF core particles. The relative reaction kinetics of TEOS and APF polymers is used to control the curvature of core particles and the growth pattern of the silica spikes, which are easily realized by the delay addition of TEOS. Specifically, the delay addition of TEOS allows a longer reaction time for the condensation of APF oligomers, which generate APF core particles with larger sizes (Scheme 1b and c). Interestingly, it is noted that the curvature of the core particles decreases with the increase of core sizes, and the growth behavior of silica spike structures are modulated accordingly. Since the silica spike structures grow perpendicularly on the core surface, the angle of the growth direction between each adjacent silica spike structures decreases along with the curvature of the core particles, and the growth behavior of silica spike structures changes from central radial behavior to para-parallel behavior. As a result, the configuration of silica spike structures can be controlled and the porous characteristics of MCSs are modulated from non-uniform to relative uniform arranged mesopores.

**Figure 3:**
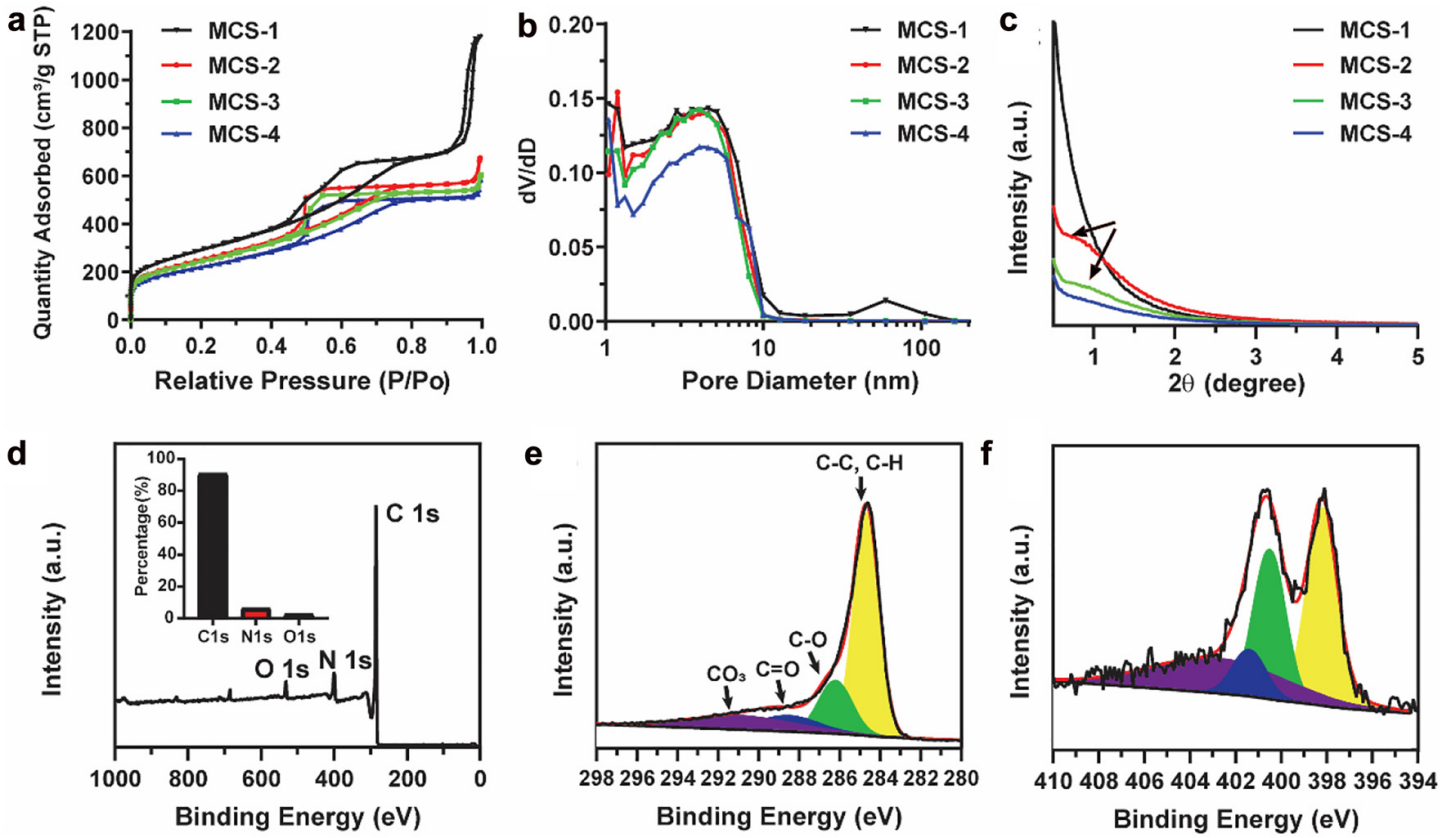
N_2_ adsorption–desorption isotherms (a), pore size distribution curves (b) and small angle X-ray diffraction patterns (c) of MCS-1, MCS-2, MCS-3, and MCS-4. XPS of MCS-3 nanoparticles: The survey spectrum (d), high-resolution spectra of C1s (e) and N1s (f). The inset in (d) is the corresponding content of each element.

In order to explore the textural properties and pore characteristic of MCSs, nitrogen sorption analysis was conducted. As shown in Figure 3a, typical IV isotherms are observed for MCS-1, MCS-2, MCS-3, and MCS-4, which indicate the existence of mesopores in these MCSs. Moreover, all these MCSs possess high specific surface area from 777.2 to 1035.3 m^2^/g (gradually decrease from MCS-1 to MCS-4, Table S1). Besides, MCS-1 has the highest pore volume of 1.81 cm^3^/g, which is much higher than that of other MCSs (MCS-2, 0.96 cm^3^/g, MCS-3, 0.87 cm^3^/g, MCS-4, 0.82 cm^3^/g). It is interesting to notice that these MCSs has similar pore sizes of ∼4.2–4.5 nm (Figure 3b), which is due to the fact that the porous structure of MCSs are all created by the presence of silica spike structures formed by the *in situ* generated silica primary particles. Since the synthetic conditions are similar for these MCSs, the size of the *in situ* generated silica primary particles and their condensation behavior are almost the same.

The pore characteristics were further evaluated by small angle X-ray diffraction measurements. The measurements were conducted in the angle range of 0.5–5°, which allowed the evaluation of the pore properties in the nanometer scale. As shown in Figure 3c, no diffraction peak is observed for MCS-1, indicating the non-uniform pore channels. With the increase of delay addition time interval, a broad peak is observed for MCS-2 and MCS-3. When further increasing the delay addition time interval, the broad peak almost disappears. The presence of the broad peak in the small angle X-ray diffraction patterns of MCS-2 and MCS-3 indicates the presence of mesopores and the alignment of the pore channels become relative uniform compared with that of MCS-1. The above observation further demonstrates that the as developed surfactant-free delay addition strategy is successful in controlling the pore structure characteristics from non-uniform to relative uniform alignment. As for the disappearance of the broad peak in MCS-4, it can be explained by comparing the structural and textural property between MCS-3 and MCS-4. It is noted that MCS-4 has larger core size than MCS-3, and these two nanoparticles have similar overall sizes (Figure 1o and p), therefore, MCS-4 has shorter range of pore channels, which may cause the decrease of the uniformity of pore channels in MCS-4 and the disappearance of the broad peak. The relative uniform pore arrangement of MCS-3 is further demonstrated by TEM characterizations shown in Figure S2, which clearly show that the relative uniform pore channels are arranged along the central core (indicated by the red lines) with dependent on the curvature of the cores. Moreover, the surface states of MCSs are investigated by X-ray photoelectron spectroscopy (XPS). As shown in Figures 3d and S3, the survey spectra of MCSs exhibit characteristic peaks for C1s, N1s, and O1s, and the related content of each element is presented in the inset. For example, MCS-3 is composed of 90.7% of C, 6.5% of N, and 2.8% of O, which is similar compared with other MCSs (Figure S4). No observable signal peak can be found at 100 eV in the survey spectrum, which indicates the *in situ* generated silica component can be completely removed through the HF etching process. Furthermore, the high resolution of C1s XPS data (Figure 3e) for MCS-3 reveals that the surface of the nanoparticles is mainly composed of C—C or C—H species (284.8 eV), together with lower concentrations of C—O (286.2 eV) and O—C=C (288.5 eV) [29]. The spectrum of N1s (Figures 3f and S3) reveals four nitrogen species, i.e., pyridinic N (398.4 eV), pyrrolic/pyridonic N (400.1 eV), graphitic-type quaternary N structure (401.1 eV), and oxidized pyridinic N (402.8 eV), which are typical nitrogen species observed in nitrogen doped carbons [30, 31].

From the above characterizations, it is noted that the MCSs exhibit high surface area and nitrogen element doped compositions, which are favor for advanced cargo molecules adsorption and as delivery vectors in various biomedical applications. Moreover, the uniformity of pore channels in mesoporous materials can greatly influence their performances as well [32]. The uniform arranged pore channels provide a relative regulated diffusion path for loaded guest molecules, which can be developed as controlled drug delivery vectors with advanced loading and release capability for biomedical applications. As a proof of concept, the obtained MCS-3 with the most relative uniform arranged pore channels is further applied to nanomedicine for photothermal triggered drug release.

**Figure 4:**
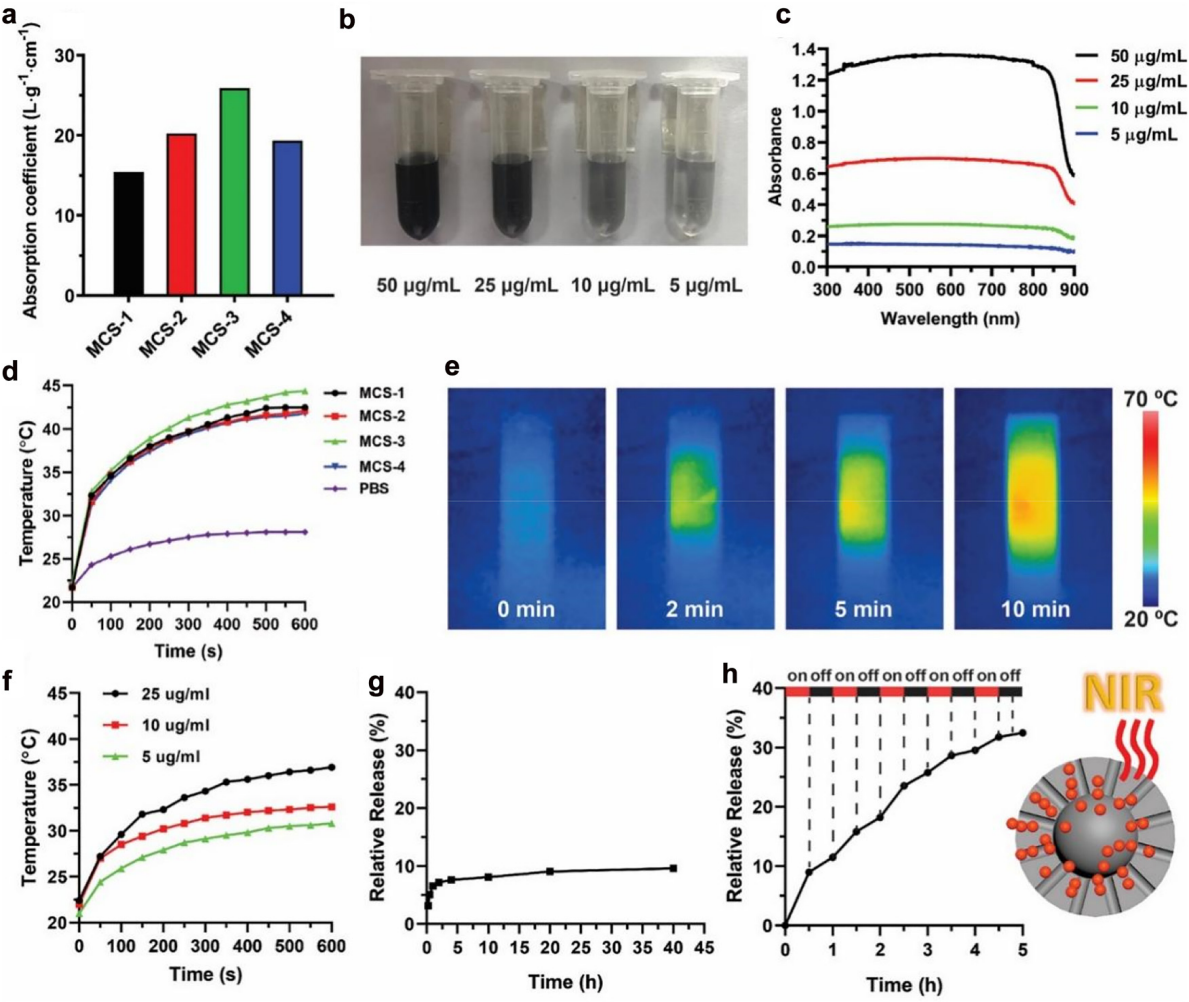
Calculated absorption coefficient for MCS-1, MCS-2, MCS-3, and MCS-4 (a). Solutions with various concentrations (b) and corresponding UV–Vis absorption spectra (c) for MCS-3. Photothermal effect of MCSs solutions (50 μg/mL) upon an NIR irradiation at 808 nm with power density of 1 W cm^−2^ (d). IR thermal images of MCS-3 solution upon NIR irradiation at 808 nm (e). Photothermal effect of MCS-3 solution upon NIR irradiation at 808 nm (1 W cm^−2^) at different concentrations (f). Free diffusion release of DOX from MCS-3 (g). Cumulative release profiles of NIR irradiation induced DOX release from MCS-3 (h).

We first evaluate the near-infrared photoresponse capability of MCSs. The UV-Vis-NIR absorption spectra are measured to evaluate the absorption property of MCSs with various concentrations in the wavelength range of 300–900 nm (Figures 4 and S5). The MCSs exhibit a broad absorption band in a wide wavelength range of 300–900 nm, which are different compared with other porous carbon materials as most of these carbon spheres only exhibit strong absorption in UV region and negligible absorption in NIR region [33, 34]. The absorption coefficients of the MCSs (Figure 4a) are calculated to be 15.43 Lg^−1^cm^−1^ (MCS-1), 20.24 Lg^−1^cm^−1^ (MCS-2), 25.88 Lg^−1^cm^−1^ (MCS-3), and 19.34 Lg^−1^cm^−1^ (MCS-4) at wavelength of 808 nm, where MCS-3 exhibit the highest absorption coefficient compared with other MCSs. It is noted that all the MCSs are well dispersed in PBS solution with concentration ranging from 5 to 50 μg/mL without precipitation (Figures 4b and S6), and all the MCSs exhibit concentration dependent absorption capability (Figure 4c). The above observations demonstrate that the near-infrared photoresponse capability of MCSs is highly related with the pore structures, which can be easily modulated by our synthetic approach.

To evaluate the photothermal conversion performance of MCSs, the as-prepared four MCSs are first dissolved in PBS solution with a concentration of 50 μg/mL. The solutions are individually exposed to NIR laser irradiation at a wavelength of 808 nm (1 W/cm^2^) for up to 10 min according to literature reports [35–37]. As shown in Figure 4d, the MCSs solutions exhibit heat generation property under NIR laser irradiation, where temperature increases by about 10 °C within 1 min, and the solutions eventually reach up to ∼41 °C for MCS-1, MCS-2 and MCS-4. MCS-3 exhibits the highest final temperature of 44 °C. The high temperature increase is not only related with the carbon-based material but also with the doped nitrogen species during the fabrication process [37]. In comparison, the temperature of pure PBS solution increase by only 7 °C. The MCSs are able to efficiently convert NIR irradiation into heat mainly due to the strong absorption in the NIR region, consistent with previous absorption spectra (Figure 4c). Among these MCSs, MCS-3 exhibit the highest absorption coefficient and photothermal conversion performance, which indicate the potential application of MCS-3 as a NIR responsive intelligent drug delivery vector. Moreover, the advanced photothermal conversion capability of MCS-3 is further characterized by IR thermal imaging. As shown in Figure 4e, rapid photothermal heating is observed upon NIR irradiation, and high temperature is reached after 10 min of NIR irradiation. The strong heat generation property of MCS-3 can still be observed under various lower concentrations (5 μg/mL, 10 μg/mL, and 25 μg/mL) (Figure 4f), and the photothermal conversion curves of MCS-3 show a distinct concentration-dependent heating effect. The NIR irradiation conversion capability of MCSs is comparable with other widely used photothermal materials, including black phosphorus [38–40], borophene [41], molybdenum dioxide [42, 43], and carbon dots [44], which indicate the biophotonic carbon spheres are promising NIR irradiation conversion agent [45, 46].

Then, the photothermal conversion capability of MCS-3 is applied in a NIR irradiation-controlled drug release assay. We first evaluate the loading performance of MCS-3 with comparison to other MCSs, and it is shown that MCS-3 exhibit high load capability towards DOX, similar with other MCSs (Figure S7). The advanced loading performances of MCSs may be related with the strong adsorption capability of carbon materials as well as the incorporation of nitrogen species which are benefit for increasing the adsorption capacity for adsorbates [47, 48]. It is know that the pore structures have great influences on the release of loaded DOX molecules. More uniform arranged pore channels can benefit the direct release of DOX, and relative long pore channels can avoid the random release of loaded cargo molecules, which provide better controlled release performances. Compared with MCS-1, MCS-2 and MCS-4, MCS-3 has the most relative uniform arranged pore channels as well as the relative long pore channels, which further suggests that MCS-3 is a better candidate as controlled drug delivery vector than carbon spheres with other structures.

Afterwards, the release profile of DOX from MCS-3 is measured. As shown in Figure 4g, the release of DOX from MCS-3 nanoparticles is relative slow under natural condition without NIR irradiation, and it can be seen that only ∼10% of the loaded DOX is released within 40 h. On the other hand, an enhanced DOX release behavior is observed when MCS-3 solution is exposed to 808 nm NIR irradiation (Figure 4h). As a result, an accumulative release of ∼33% is observed within only 5 h after a few times of 808 nm NIR irradiation, which is significantly higher than the free diffusion releasing. The stepwise triggered enhanced DOX release under NIR irradiation can be ascribed to the advanced photothermal conversion capability of MCS-3. The heat generated within the inner side of the nanoparticles create temperature gradient and thermal conduction from the inner side to the outer surface, which promote the release of loaded DOX molecules. Furthermore, the relative uniform arranged pore channels of MCS-3 can further promote the release performances as well. The relative uniform arranged pore structures provide para-parallel aligned straight pores which facilitate the diffusion of loaded DOX molecules, especially under the thermal gradient.

**Figure 5:**
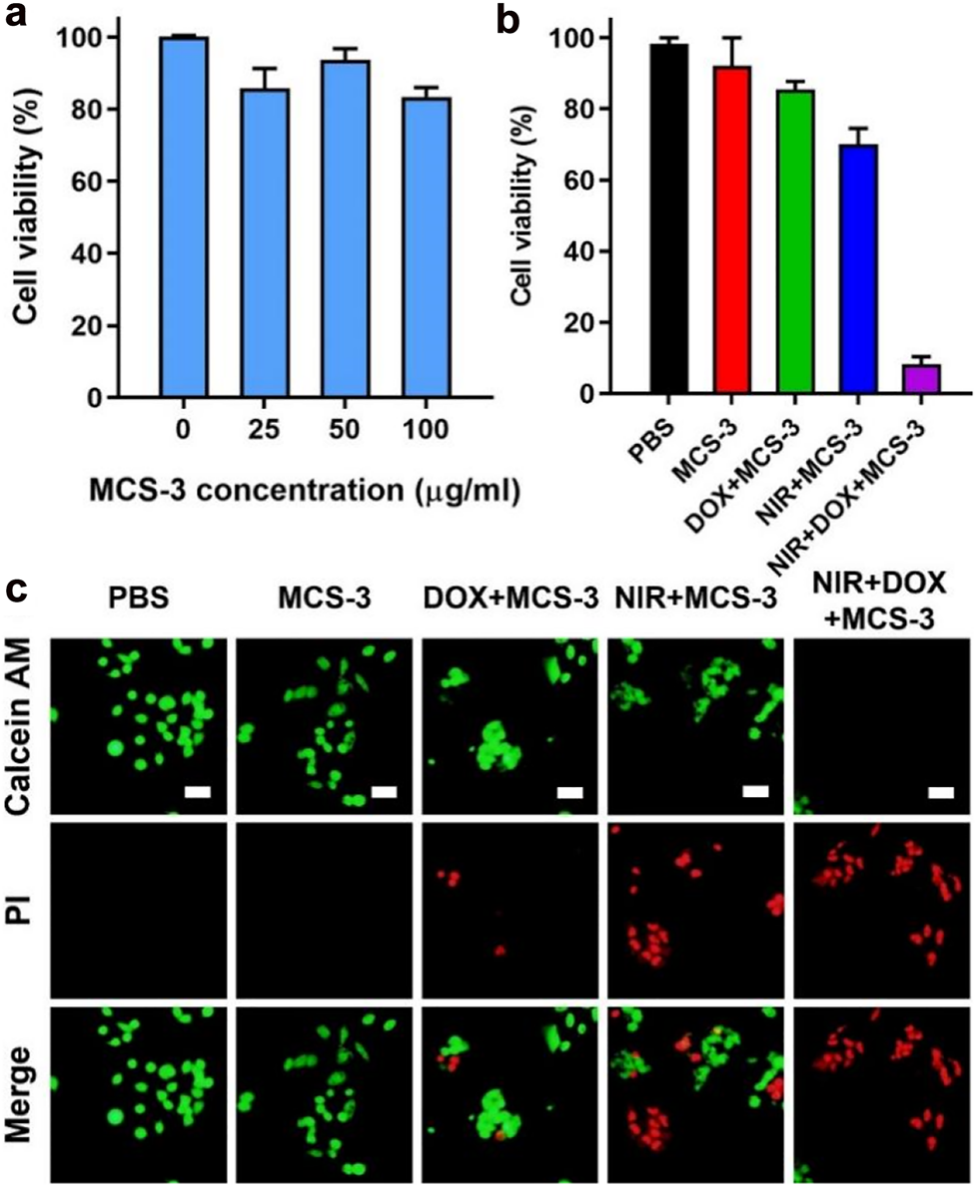
CCK-8 assay results of KHOS cells incubated with MCS-3 under various concentrations ranging from 0 to 100 μg/mL for 24 h (a). *In vitro* cancer cell death after NIR photothermal ablation under different formulations (50 μg/mL) (b). Representative fluorescent microscopic images with Calcein AM/PI staining of live cells (green) and dead cells (red) from the corresponding formulations (c). All scale bars are 25 μm.

At last, we evaluate the cytotoxicity, *in vitro* photothermal effect and controlled drug release capability of MCS-3 in KHOS cells by using the standard CCK-8 assay. As shown in Figure 5a, MCS-3 exhibit low cytotoxicity when the cells are incubated with high nanoparticle concentrations up to 100 μg/mL for 24 h, indicating the relative good biocompatibility of MCS-3. Furthermore, the photothermal conversion capability and the corresponding advanced NIR controlled drug release capability of MCS-3 are demonstrated *in vitro* to show its potential application as a multifunctional drug delivery platform. Effect of the photothermal and NIR controlled drug release are examined by Calcein AM/PI staining assay. As shown in Figure 5b, statistics results shown that ∼20% cells are killed in the DOX loaded MCS-3 group and ∼30% cells are killed after 808 nm NIR light irradiation, respectively. Moreover, for the NIR light irradiation treated DOX loaded MCS-3 group, it is observed that ∼90% cells are killed, indicating the efficient killing of cancer cells. It is reasoned that the NIR light irradiation can result in the following two effects. On one hand, the photothermal effect of MCS-3 can increase the temperature of the cancer cell local environment and kill the cells. On the other hand, the photothermal effect can stimulate the release of DOX from MCS-3 matrix. As a result of the combined functions, the killing effect is largely enhanced. Furthermore, representative corresponding Calcein AM/PI staining fluorescent images are presented in Figure 5c showing the live cells (green) and dead cells (red), which is consistent with the statistics results in Figure 5b. The above results demonstrate the feasibility of using MCS-3 as NIR light controlled drug delivery vector and advanced photothermal agent.

## 3 Conclusions

In summary, we have developed an easy controllable surfactant-free fabrication approach to synthesis MCSs with tunable pore structure characteristics. The porous nature of MCSs is controlled from non-uniform central radial pore channels to para-parallel pore channels with relative uniform arranged mesoporous structures simply by modulating the reaction kinetics of the polymerization systems. The obtained nanoparticles exhibit uniform particle sizes and advanced photothermal conversion capability. The near-infrared photoresponse capability is demonstrated related with the pore structures of the MCSs. Moreover, the enhanced near-infrared photoresponse capability is used to control the release of loaded cargo molecules. Together with the high surface area and well controlled uniformity of the arrangement of mesopore structures, the MCS-3 nanoparticles can be used as a novel drug delivery system. The advanced adsorption capability of MCSs can restrict the leakage of loaded drug molecules in natural condition, while the near-infrared photoresponse capability allows the release in a controlled stimuli-responsive way. As last, we have demonstrated the application of MCS-3 as dual function drug delivery platform *in vitro*. We believe our synthetic strategy can lead to future development of surfactant-free method to control the uniformity of pore channel. The MCSs developed in this work can also be used as versatile platforms for more biomedical applications.

## Supplementary Material

Supplementary Material Details

## References

[1] Li C., Li Q., Kaneti Y. V. (2020). Self-assembly of block copolymers towards mesoporous materials for energy storage and conversion systems. Chem. Soc. Rev..

[2] Li W., Liu J., Zhao D. (2016). Mesoporous materials for energy conversion and storage devices. Nat. Rev. Mater..

[3] Sun H., Zhu J., Baumann D. (2019). Hierarchical 3D electrodes for electrochemical energy storage. Nat. Rev. Mater..

[4] Chen S. Z., Deng Y. X., Cao X. H. (2019). Exploring high-performance anodes of Li-ion batteries based on the rules of pore-size dependent band gaps in porous carbon foams. J. Mater. Chem. A.

[5] Matsui T., Tanaka S., Miyake Y. (2013). Correlation between the capacitor performance and pore structure of ordered mesoporous carbons. Adv. Powder Technol..

[6] Zhang H. W., Noonan O., Huang X. D. (2016). Surfactant-free assembly of mesoporous carbon hollow spheres with large tunable pore sizes. ACS Nano.

[7] Zhang H., Yu M., Song H. (2015). Self-organized mesostructured hollow carbon nanoparticles via a surfactant-free sequential heterogeneous nucleation pathway. Chem. Mater..

[8] Benzigar M. R., Talapaneni S. N., Joseph S. (2018). Recent advances in functionalized micro and mesoporous carbon materials: synthesis and applications. Chem. Soc. Rev..

[9] Wei J., Zhou D., Sun Z. (2013). A controllable synthesis of rich nitrogen-doped ordered mesoporous carbon for CO_2_ capture and supercapacitors. Adv. Funct. Mater..

[10] Yang X., Lu P., Yu L. (2020). An efficient emulsion-induced interface assembly approach for rational synthesis of mesoporous carbon spheres with versatile architectures. Adv. Funct. Mater..

[11] Ma T. Y., Liu L., Yuan Z. Y. (2013). Direct synthesis of ordered mesoporous carbons. Chem. Soc. Rev..

[12] Tian H., Wang T., Zhang F. (2018). Tunable porous carbon spheres for high-performance rechargeable batteries. J. Mater. Chem. A Mater. Energy. Sustain..

[13] Kortesuo P., Ahola M., Karlsson S. (2000). Silica xerogel as an implantable carrier for controlled drug delivery-evaluation of drug distribution and tissue effects after implantation. Biomaterials.

[14] Saha D., Warren K. E., Naskar A. K. (2014). Soft-templated mesoporous carbons as potential materials for oral drug delivery. Carbon.

[15] Vallet-Regi M., Balas F., Arcos D. (2007). Mesoporous materials for drug delivery. Angew. Chem. Int. Ed..

[16] Song S. W., Hidajat K., Kawi S. (2005). Functionalized SBA-15 materials as carriers for controlled drug delivery: influence of surface properties on matrix-drug interactions. Langmuir.

[17] Slowing I., Trewyn B. G., Lin V. S. Y. (2006). Effect of surface functionalization of MCM-41-type mesoporous silica nanoparticles on the endocytosis by human cancer cells. J. Am. Chem. Soc..

[18] Mellaerts R., Aerts C. A., Van Humbeeck J. (2007). Enhanced release of itraconazole from ordered mesoporous SBA-15 silica materials. *Chem. Commun.*.

[19] Meng L., Liu Z., Fang W., Xuan J. (2017). The influence of the type of N dopping on the performance of bifunctional N-doped ordered mesoporous carbon electrocatalysts in oxygen reduction and evolution reaction. J. Energy Chem..

[20] Tang J., Liu J., Li C. L. (2015). Synthesis of nitrogen-doped mesoporous carbon spheres with extra-large pores through assembly of diblock copolymer micelles. Angew. Chem. Int. Ed..

[21] Liu J., Yang T. Y., Wang D. W. (2013). A facile soft-template synthesis of mesoporous polymeric and carbonaceous nanospheres. Nat. Commun..

[22] Ji X., Lee K. T., Nazar L. F. (2009). A highly ordered nanostructured carbon-sulphur cathode for lithium-sulphur batteries. Nat. Mater..

[23] Guan B. Y., Yu L., Lou X. W. (2016). Formation of asymmetric bowl-like mesoporous particles via emulsion-induced interface anisotropic assembly. J. Am. Chem. Soc..

[24] Choi Y. J., Kim H. K., Lee S. W. (2017). Surfactant-free synthesis of a nanoperforated graphene/nitrogen-doped carbon nanotube composite for supercapacitors. J. Mater. Chem. A.

[25] Mohammadnezhad G., Akintola O., Plass W., Steiniger F., Westermann M. (2016). A facile, green and efficient surfactant-free method for synthesis of aluminum nanooxides with an extraordinary high surface area. Dalton Trans..

[26] Strubel P., Thieme S., Biemelt T. (2015). ZnO hard templating for synthesis of hierarchical porous carbons with tailored porosity and high performance in lithium-sulfur battery. Adv. Funct. Mater..

[27] Fei R. X., Wang H. W., Wang Q. (2020). In situ hard-template synthesis of hollow bowl-like carbon: a potential versatile platform for sodium and zinc ion capacitors. Adv. Energy Mater..

[28] Feng Y., He T., Alonso-Vante N. (2008). In situ free-surfactant synthesis and ORR- electrochemistry of carbon-supported Co_3_S_4_ and CoSe_2_ nanoparticles. Chem. Mater..

[29] Zhou L., Jing Y., Liu Y. (2018). Mesoporous carbon nanospheres as a multifunctional carrier for cancer theranostics. Theranostics.

[30] Feng S., Li W., Shi Q. (2014). Synthesis of nitrogen-doped hollow carbon nanospheres for CO_2_ capture. Chem. Commun..

[31] Xu C., Niu D., Zheng N. (2018). Facile synthesis of nitrogen-doped double-shelled hollow mesoporous carbon nanospheres as high-performance anode materials for lithium ion batteries. ACS Sustain. Chem. Eng..

[32] Li X., Zhao T., Lu Y. (2017). Degradation-restructuring induced anisotropic epitaxial growth for fabrication of asymmetric diblock and triblock mesoporous nanocomposites. Adv. Mater..

[33] Zhou L., Dong K., Chen Z. (2015). Near-infrared absorbing mesoporous carbon nanoparticle as an intelligent drug carrier for dual-triggered synergistic cancer therapy. Carbon.

[34] Wang H., Di J., Sun Y. (2015). Biocompatible PEG-chitosan@carbon dots hybrid nanogels for two-photon fluorescence imaging, near-infrared light/pH dual-responsive drug carrier, and synergistic therapy. Adv. Funct. Mater..

[35] Sun Z., Xie H., Tang S. (2015). Ultrasmall black phosphorus quantum dots: synthesis and use as photothermal agents. Angew. Chem. Int. Ed..

[36] Wang S., Shang L., Li L. (2016). Meta-organic-framework-derived mesoporous carbon nanospheres containing porphyrin-like metal centers for conformal phototherapy. Adv. Mater..

[37] Lee C., Kwon W., Beack S. (2016). Biodegradable nitrogen-doped carbon nanodots for non-invasive photoacoustic imaging and photothermal therapy. Theranostics.

[38] Zhao H., Chen H., Guo Z. (2020). In situ photothermal activation of necroptosis potentiates black phosphorus-mediated cancer photo-immunotherapy. Chem. Eng. J..

[39] Xie Z., Peng M., Lu R. (2020). Black phosphorus-based photothermal therapy with aCD47-mediated immune checkpoint blockade for enhanced cancer immunotherapy. Light Sci. Appl..

[40] Zhu Y., Xie Z., Li J. (2021). From phosphorus to phosphorene: applications in disease theranostics. Coord. Chem. Rev..

[41] Xie Z., Meng X., Li X. (2020). Two-dimensional borophene: properties, fabrication, and promising applications. Research.

[42] Lan P., Chen H., Guo Y. (2022). NIR-II responsive molybdenum dioxide nanosystem manipulating cellular immunogenicity for enhanced tumor photoimmunotherapy. Nano Lett..

[43] Guo Y., Li Y., Zhang W. (2020). Insights into the deep-tissue photothermal therapy in near-infrared II region based on tumor-targeted MoO2 nanoaggregates. Sci. China Mater..

[44] Liu H., Mo L., Chen H. (2022). Carbon dots with intrinsic bioactivities for photothermal optical coherence tomography, tumor-specific therapy and postoperative wound management. Adv. Healthc. Mater..

[45] Liu H., Chen C., Chen H. (2022). 2D-PROTACs with augmented protein degradation for super-resolution photothermal optical coherence tomography guided momentary multimodal therapy. Chem. Eng. J..

[46] Chen H., Liu Z., Jiang O. (2021). Nanocomposite of Au and black phosphorus quantum dots as versatile probes for amphibious SERS spectroscopy, 3D photoacoustic imaging and cancer therapy. Giant.

[47] Inagaki M., Toyoda M., Soneda Y., Morishita T. (2018). Nitrogen-doped carbon materials. Carbon.

[48] Gao Y., He D., Wu L. (2021). Porous and ultrafine nitrogen-doped carbon nanofibers from bacterial cellulose with superior adsorption capacity for adsorption removal of low-concentration 4-chlorophenol. Chem. Eng. J..

