# Supplementary material for: Mesoporous biophotonic carbon spheres with tunable curvature for intelligent drug delivery
Source: Nanophotonics. 2022 Oct 4;11(22):5165–75. doi: 10.1515/nanoph-2022-0523 (PMC11501982; doi:10.1515/nanoph-2022-0523)
Supplement: Supplementary file 1 — Supplementary Material Details [file j_nanoph-2022-0523_suppl_001.docx]

**Supporting Information**

Mesoporous biophotonic carbon spheres with tunable curvature for intelligent drug delivery

*Jianye Fu^a,b,c,†^, Tiankun Hui^a,†^, Dong An^b,†^, Wei Shan^a^, Guobo Chen^a^, Swelm Wageh^d^, Omar A. Al-Hartomy^d^, Bin Zhang^b^, Ni Xie^b^, Guohui Nie^b^, Jinqing Jiao^a,e,^*, Meng Qiu^a,^* and Han Zhang^b,^**

a Key Laboratory of Marine Chemistry Theory and Technology (Ocean University of China), Ministry of Education, Qingdao, 266100, China

b Collaborative Innovation Center for Optoelectronic Science & Technology, International Collaborative Laboratory of 2D Materials for Optoelectronics Science and Technology of Ministry of Education, Institute of Microscale Optoelectronics, College of Physics and Optoelectronic Engineering, Shenzhen University, Shenzhen 518060, P. R. China

c College of Chemistry and Chemical Engineering, China University of Petroleum, Qingdao 266555, China

d Department of Physics, Faculty of Science, King Abdulaziz University, Jeddah 21589, Saudi Arabia

e State Key Laboratory of Safety and Control for Chemicals, SINOPEC Research Institute of Safety Engineering, Qingdao, 266071, China

† These authors contributed equally to this work.

Corresponding Author

Jinqing Jiao, Email: jjq469@163.com

Meng Qiu, Email: mengqiu@ouc.edu.cn

Han Zhang, Email: hzhang@szu.edu.cn


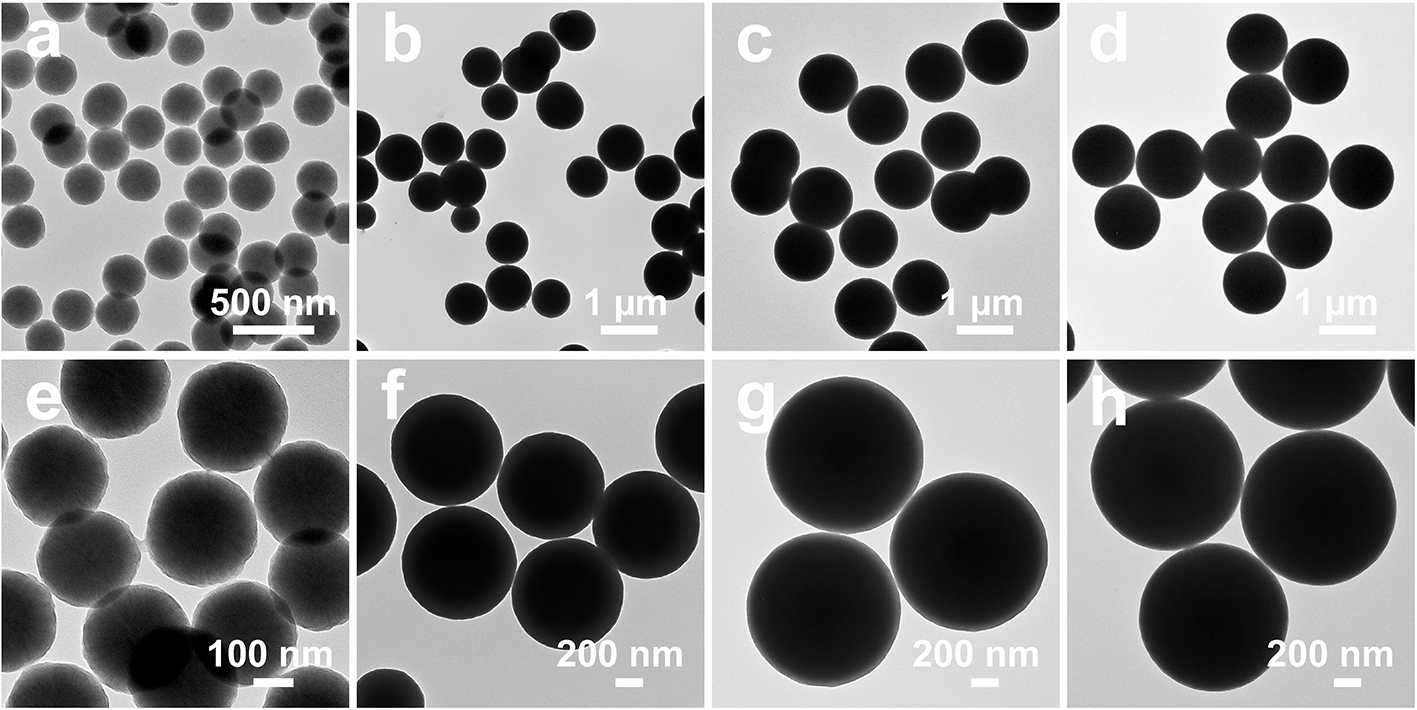


**Figure S1**. Low (a-d) and high (e-h) magnification TEM images of the as-synthesized APF/silica composite spheres. MCS-1 composites (a, e), MCS-2 composites (b, f), MCS-3 (c, g) composites and MCS-4 composites (d, h).


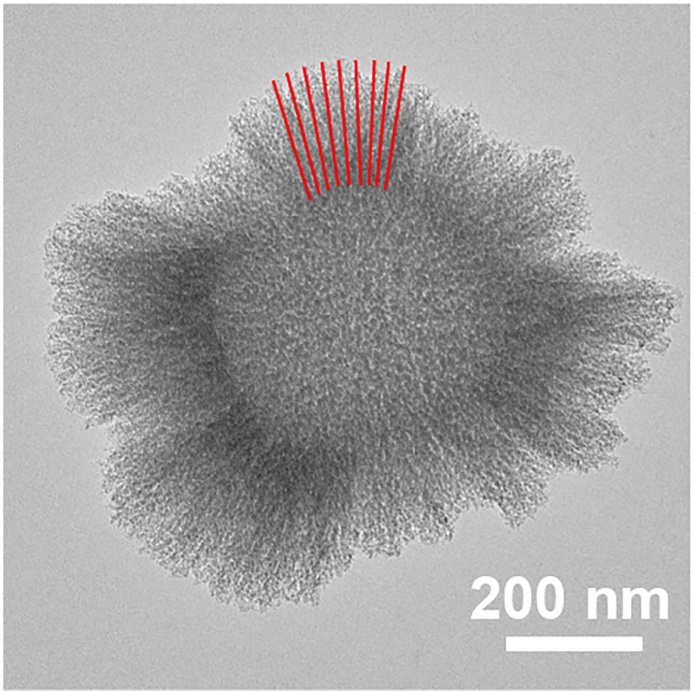


**Figure S2**. Representative TEM image of the silica template fragments of MCS-3.


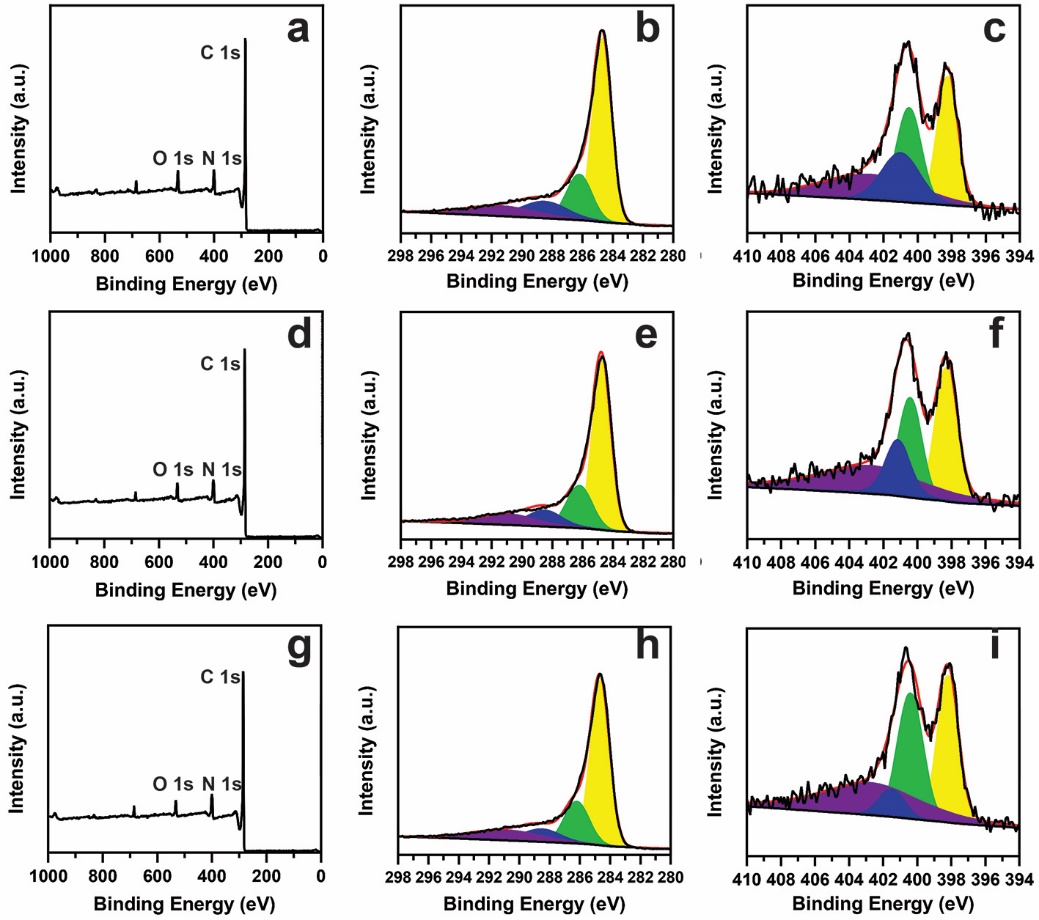


**Figure S3.** XPS of MCS-1 (a, b, c), MCS-2 (d, e, f) and MCS-4 (g, h, i): the survey spectrum (a, d, g), high-resolution spectra of C1s (b, e, h) and N1s (c, f, i).


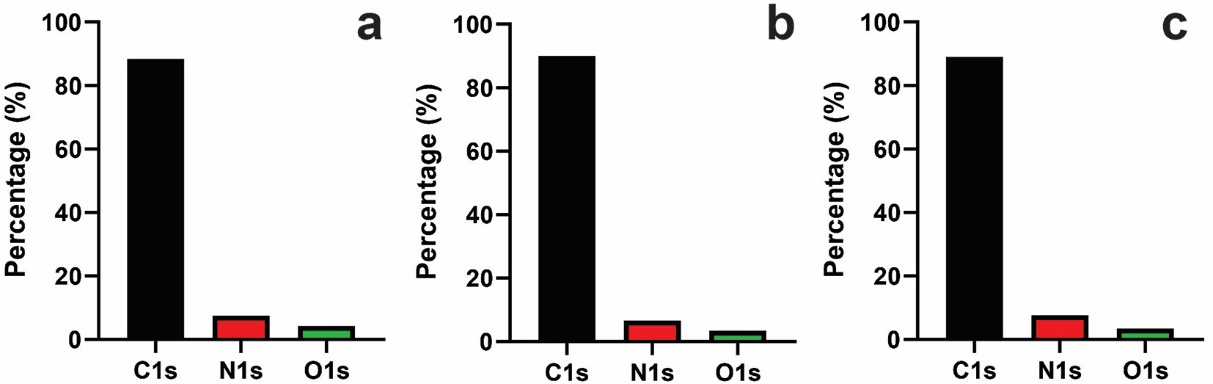


**Figure S4.** XPS measurement of each element content in MCS-1 (a), MCS-2 (b) and MCS-4 (c).


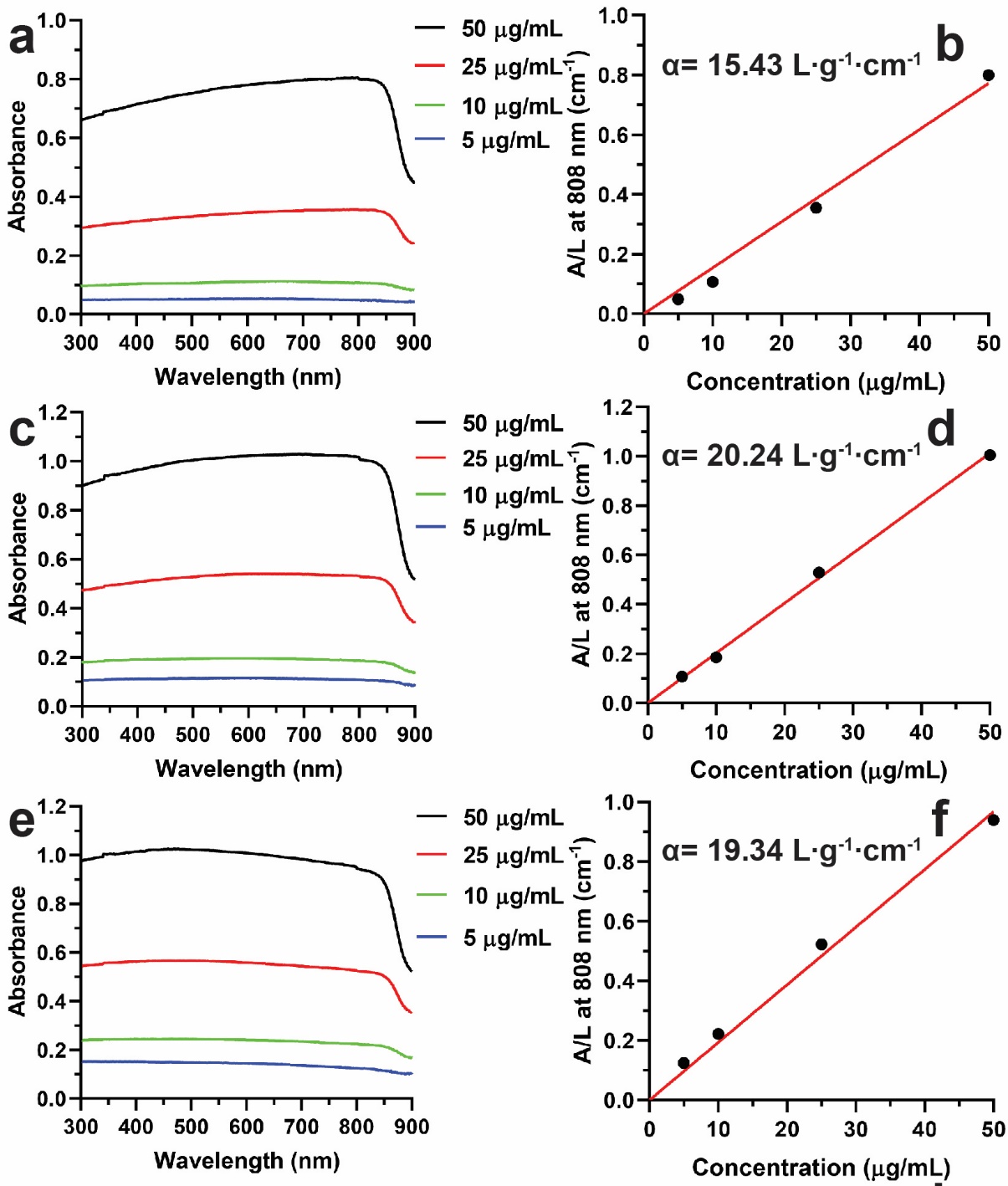


**Figure S5**. UV-Vis absorption spectra for MCSs (a, MCS-1; c, MCS-2; e, MCS-4) dispersed in PBS solution at concentrations from 5 to 50 μg/mL and corresponding molar absorption coefficient (b, d, f) calculated from the absorbance at 808 nm, respectively.


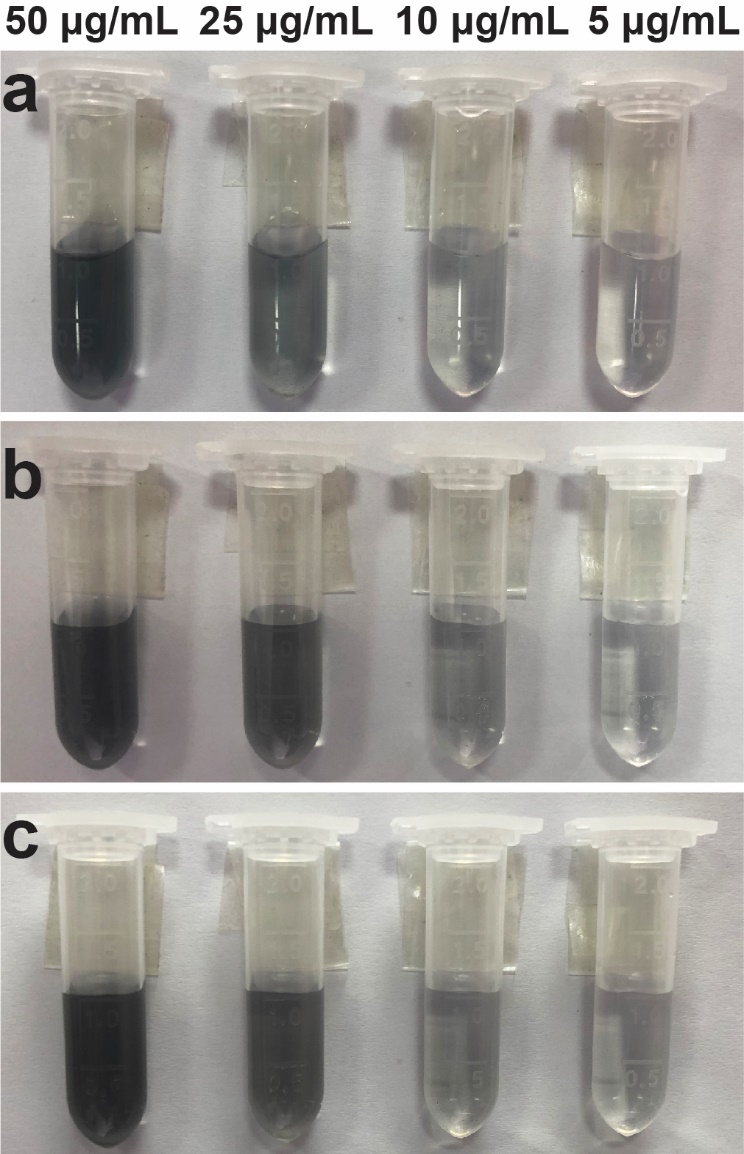


**Figure S6.** Digital images of MCS-1 (a), MCS-2 (b) and MCS-4 (c) dispersed in PBS solution with various concentrations.


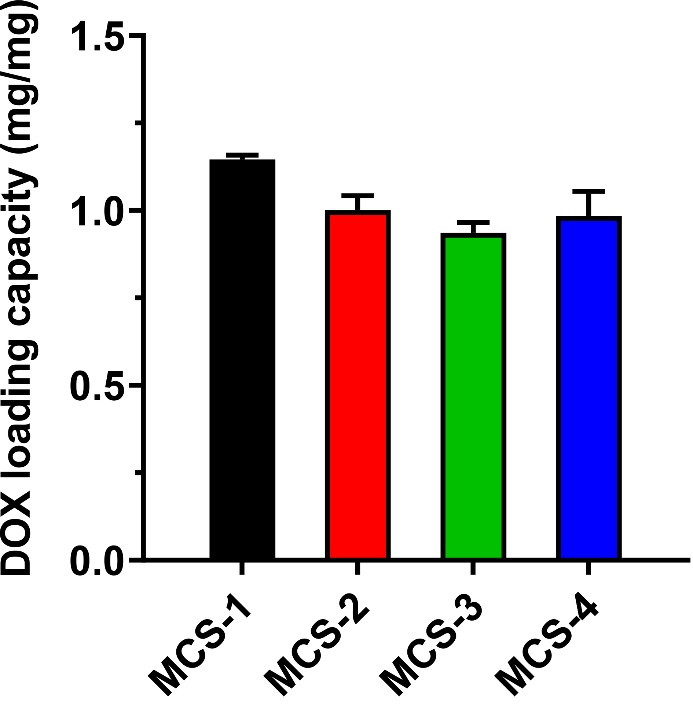


**Figure S7.** DOX loading capacity of MCS-1, MCS-2, MCS-3 and MCS-4.

**Table S1.** Textural and structural characteristics of MCSs

| Samples | S_BET_ (m^2^ g^-1^) | V_p_ (cm^3^ g^-1^) | D_p_ (nm) |
| --- | --- | --- | --- |
| MCS-1 | 1035.3 | 1.81 | 4.5 |
| MCS-2 | 896.2 | 0.96 | 4.3 |
| MCS-3 | 837.2 | 0.87 | 4.2 |
| MCS-4 | 777.2 | 0.82 | 4.2 |
